# Hypoalbuminaemia as a Prognostic Biomarker of First-Line Treatment Resistance in Metastatic Non-small Cell Lung Cancer

**DOI:** 10.3389/fnut.2021.734735

**Published:** 2021-10-01

**Authors:** Mark Stares, Amanda Swan, Kirsten Cumming, Tze-En Ding, James Leach, Cory Stratton, Findlay Thomson, Colin Barrie, Kirsty MacLennan, Sorcha Campbell, Tamasin Evans, Aisha Tufail, Stephen Harrow, Melanie MacKean, Iain Phillips

**Affiliations:** ^1^Edinburgh Cancer Centre, Edinburgh, United Kingdom; ^2^Edinburgh Cancer Research UK Centre, Edinburgh, United Kingdom

**Keywords:** albumin (ALB), non-small cell lung (NSCLC), prognostic biomarker, systemic anticancer therapy, targeted therapies, immunotherapies, systemic inflammation, cachexia

## Abstract

**Introduction:** Despite significant advances in systemic anticancer therapy (SACT) for non-small cell lung cancer (NSCLC), many patients still fail to respond to treatment or develop treatment resistance. Albumin, a biomarker of systemic inflammation and malnutrition, predicts survival in many cancers. We evaluated the prognostic significance of albumin in patients receiving first-line targeted therapy or immunotherapy-based SACT for metastatic NSCLC.

**Methods:** All patients treated with first-line targeted therapy or immunotherapy-based SACT for metastatic NSCLC at a regional Scottish cancer centre were identified. Serum albumin at pre-treatment, after 12-weeks of treatment, and at the time of progressive disease were recorded. The relationship between albumin (≥ 35g/L v <35g/L) and overall survival (OS) was examined.

**Results:** Data were available for 389 patients of both targeted therapy cohort (*n* = 159) and immunotherapy-based therapy cohort (*n* = 230). Pre-treatment albumin was predictive of OS in each cohort at HR1.82 (95%CI 1.23–2.7) (*p* =0.003) and HR2.55 (95%CI 1.78–3.65) (*p* < 0.001), respectively. Pre-treatment albumin <35 g/L was associated with a significantly higher relative risk of death within 12 weeks in each cohort at RR9.58 (95%CI 2.20–41.72, *p* = 0.003) and RR3.60 (95%CI 1.74–6.57, *p* < 0.001), respectively. The 12-week albumin was predictive of OS in each cohort at HR1.88 (95%CI 1.86–4.46) (*p* < 0.001) and HR2.67 (95%CI 1.74–4.08) (*p* < 0.001), respectively. 46 out of 133 (35%) evaluable patients treated with targeted therapy and 43 out of 169 (25%) treated with immunotherapy-based therapy crossed over albumin prognostic groups between pre-treatment and 12-week. The prognostic value of 12-week albumin was independent of pre-treatment albumin status. A majority of patients had albumin <35g/L at the time of progressive disease when it was also predictive of survival following progressive disease at HR2.48 (95%CI 1.61–3.82) (*p* < 0.001) and HR2.87 (95%CI 1.91–4.31) (*p* < 0.001) respectively).

**Conclusions:** Albumin is a reliable prognostic factor in patients with metastatic NSCLC, predicting survival independent of the class of drug treatment at various time points during the patient journey. Tracking albumin concentrations during systemic therapy may indicate disease activity or treatment response over time.

## Introduction

Lung cancer is the second most common cancer worldwide and accounts for 25% of all cancer deaths (1). Non-small cell lung cancer (NSCLC) represents approximately 85% of all cases. Unfortunately, the majority present with metastatic disease for whom the prognosis is often poor (2). Historically, NSCLC was considered as a single disease, and patients with metastatic disease were treated with cytotoxic chemotherapy. Advances in our understanding of the biology of NSCLC have defined molecular subtypes of NSCLC with distinct clinical characteristics and therapeutic options. Small molecule tyrosine kinase inhibitor (TKI) “*targeted therapies”* are available for the 10–15% of patients with NSCLC harbouring epidermal growth factor receptor (EGFR) mutations, i.e., erlotinib, gefitinib, afatinib, and osimertinib, and the 5–6% harbouring anaplastic lymphoma kinase (ALK) gene rearrangements or c-ros oncogene 1 (ROS1) mutations, i.e., crizotinib and alectinib (3–5). In patients without such actionable mutations, the monoclonal antibody “*immunotherapy”* agents which inhibit immune checkpoints, i.e., pembrolizumab, nivolumab, atezolizumab, and durvalumab, have emerged as standard-of-care treatment (6–12).

Clinical assessment of the fitness of a patient, using tools such as the Eastern Co-Operative Group Performance Status (PS), and consideration of the comorbidities of a patient determine whether it is appropriate to treat an individual with these agents. The identification of objective prognostic factors that predict response to systemic therapy in NSCLC would be invaluable tools for clinical decision-making. In particular, factors that identify those that are least likely to respond to systemic therapy or that predict the development of treatment resistance may allow patients to pursue other treatment options or facilitate early referral to palliative care and appropriate arrangements for end of life care.

Inflammation plays a key role in the development, survival, and progression of cancer (13). In addition to interactions at the level of the tumour microenvironment, systemic inflammatory effects are observed and frequently measured by routine clinical tests such as serum albumin. Historically, albumin and other serum proteins have been widely used by physicians as biomarkers of malnutrition (14–16). However, serum levels of this cheap, readily available blood biomarker are significantly depressed as a result of inflammation through decreased synthesis and increased catabolism (16, 17). As such, albumin is considered a biomarker of cachexia, a state characterised by weight loss, systemic inflammation and reduced functional status and associated with poor prognosis in many diseases (18–20). Indeed, hypoalbuminaemia is a recognised poor prognostic factor in cancer, both individually and as a core component of composite prognostic scores (15, 21–24). However, much of the established literature for the use of albumin as a biomarker in NSCLC pre-dates the routine use of immunotherapy and molecular profile directed targeted therapy treatment in clinical practise (24–27).

The Edinburgh Cancer Centres provide regional cancer services in Scotland, UK, serving a population of approximately 1.5 million. We utilised real-world experience to examine the prognostic value of serum albumin concentration (g/L) in patients with metastatic NSCLC receiving first-line targeted therapy or immunotherapy-based systemic anticancer therapy (SACT).

## Materials and Methods

### Study Population

All patients being treated with the first-line SACT for metastatic NSCLC by the Edinburgh Cancer Centre Lung Cancer Service, NHS Lothian, between June 2016 and January 2021 were identified from the electronic prescribing record. Eligible patients were 18 years or over, had a pathological diagnosis of NSCLC, were felt to be fit for systemic anticancer therapy (SACT) when assessed in the pre-treatment specialist oncology clinic, and had received at least one dose of targeted therapy or immunotherapy-based SACT regimen.

### Procedure and Assessment

Patient demographics and pathological data were recorded. Serum albumin was recorded at pre-treatment, within 14 days prior to cycle 1, day 1 (C1D1) SACT; 12-weeks (+/−14 days 12 weeks after C1D1 SACT); progressive disease (PD) (+/−14 days at the time of radiological or clinical evidence of disease progression prompting cessation of treatment.

All data were collected as part of routine oncology work-up in keeping with the standard of care. No patient identifiable data were used. The presented work was in accordance with guidelines from the Academic and Clinical Central Office for Research and Development (ACCORD, NHS Lothian, and the University of Edinburgh) and specific consent was not required. Since the study was not designed to test a formal hypothesis, a sample size calculation was not required. All patients treated during the aforementioned time period were assessed.

### Statistical Analysis

Overall survival (OS), defined as the number of months from C1D1 SACT until death, or censorship (May 25, 2021), if alive at follow-up date, was calculated. Additionally, PD-OS, defined as the time between PD until death, or censorship (May 25, 2021), if alive at follow-up date, was calculated. Survival curves were plotted using Kaplan–Meier methods and the log-rank test was applied. Survival analysis was carried out using Cox's proportional-hazards model, and hazard ratios were calculated. All analyses were performed in SPSS Version 24 (SPSS Inc) IBM, USA. The study adhered to the Reporting Recommendations for Tumour Marker Prognostic Studies guideline.

## Results

### Patient Characteristics

Data were available for 389 patients with a median age of 67 [interquartile range (IQR) 59–72)] and among which 223 (57%) were men. Patients were divided into 2 cohorts by treatment modality for further analysis. The first cohort involved 159 (41%) patients who received targeted therapy (Table 1), of which the majority [*n* = 132 (83%)] received an EGFR TKI. Median OS was 18.7 (IQR 8.8–38.2) months. At the time of censoring, 57 (36%) of patients were alive. The minimum and the median follow-up of survivors was 3.7 months and 16.8 months, respectively.

**Table 1:** Clinical characteristics and survival in patients with NSCLC treated with first-line targeted therapy or immunotherapy-based therapy: univariate log-rank analysis.

		**Targeted Therapy Cohort**			**Immunotherapy Cohort**		
		***n = 159***	***Overall Survival (months)***		***n* = 230**	***Overall Survival (months)***	
**All**		***n* (%)**	**Median (IQR)**	***p***	***n* (%)**	**Median (IQR)**	***p***
Age	≤ 64	69 (43)	21.9 (12.9–38.2)	***0.038***	94 (41)	10.0 (4.4–21.4)	*0.052*
	65–74	53 (33)	15.3 (6.9–39.6)		115 (50)	17.6 (6.4–n/r)	
	>74	37 (23)	12.9 (3.8–20.4)		21 (9)	13.9 (6.5–25.0)	
Sex	Female	51 (32)	19.9 (8.2–39.4)	*0.319*	115 (50)	10.8 (4.7–n/r)	*0.204*
	Male	108 (68)	17.7 (9.5–26.3)		115 (50)	17.4 (4.9–n/r)	
Histologic Subtype	Squamous	0 (0)	n/a	*n/a*	44 (19)	12.4 (4.6-n/r)	*0.600*
	Non-Squamous	159 (100)	18.7 (8.8–38.2)		186 (81)	11.0 (7.0-n/r)	
Treatment	Afatinib	54 (34)	20.2 (12.1–38.2)	***0.604***	n/a	n/a	*0.657*
	Erlotinib	62 (39)	12.0 (6.7–26.8)		n/a	n/a	
	Osimertinib	16 (10)	16.5 (4.6–39.4)		n/a	n/a	
	Alectinib	16 (10)	*n*/r		n/a	n/a	
	Crizotinib	11 (7)	17.7 (5.2–n/r)		n/a	n/a	
	Pembrolizumab	n/a	n/a		167 (73)	13.4 (4.5-n/r)	
	Chemo-immunotherapy	n/a	n/a		63 (27)	11.8 (6.4-18.9)	
ECOG Performance Status	0	30 (20)	26.8 (16.2–39.4)	***0.002***	37 (16)	n/r (10.1-n/r)	***<** **0.001***
	1	93 (58)	18.5 (11.7–43.2)		165 (72)	13.4 (5.0-n/r)	
	2+	36 (23)	7.5 (3.5–21.6)		28 (12)	6.0 (2.1-10.4)	
Albumin	≥35g/L	101 (64)	21.9 (11.7–39.4)	***0.002***	120 (52)	19.7 (9.7-n/r)	***<** **0.001***
	<35g/L	58 (36)	12.4 (4.4–21.9)		110 (48)	7.8 (2.5–20.5)	

*n/r = not reached. Bold values are statistically significant variables*.

Furthermore, 230 (59%) patients received first-line immunotherapy-based therapy (Table 1). The majority which summed up at 167 (73%) had received pembrolizumab monotherapy, reflecting the longer availability of this regimen during the time period of the study. Median OS was 12.4 (IQR 4.9–not reached) months. At the time of censoring, 102 (44%) of patients were alive. The minimum and the median follow-up of survivors was 4.8 months and 14 months, respectively.

Patients in the targeted therapy cohort were more frequently men [68% vs. 50% (*p* < 0.001)], PS2+ [23 vs. 12% (*p* = 0.004)], or pre-treatment albumin ≥35 g/L [64 vs. 52% (*p* = 0.015)]. There was no difference in PFS or OS between the two cohorts (*p* > 0.05).

### Prognostic Value of Pre-treatment Albumin

**Figure 1:**
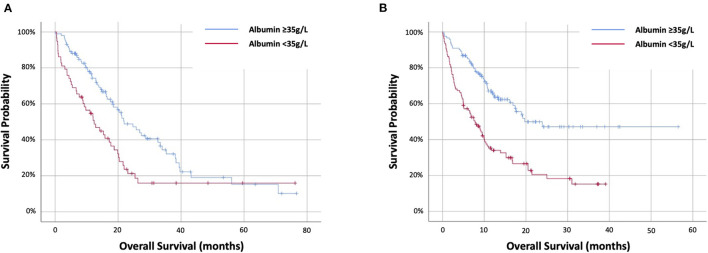
Kaplan–Meier survival curves examining the relationship between pre-treatment albumin and overall survival patients with NSCLC treated with first-line **(A)** targeted therapy or **(B)** immunotherapy-based therapy.

The prognostic value of pre-treatment albumin was evaluated. In the targeted therapy cohort age, PS and pre-treatment albumin were univariately associated with OS (log-rank) with values of *p* = 0.038, *p* = 0.002 and *p* = 0.002, respectively (Table 1). Pre-treatment albumin was predictive of OS at HR1.82 (95%CI 1.23–2.7) (*p* = 0.003) and stratified OS from 12.4 (IQR 4.4–21.9) months (<35 g/L) to 21.9 (IQR 11.7–39.4) months (≥35 g/L) (*p* = 0.002) (Figure 1A). In the immunotherapy cohort PS and pre-treatment albumin were univariately associated with OS (log-rank) with values of *p* < 0.001 and *p* < 0.001, respectively (Table 1). Pre-treatment albumin was predictive of OS at HR2.55 (95%CI 1.78-3.65) (*p* < 0.001) and stratified OS from 7.8 (IQR 2.5–20.5) months (<35 g/L) to 19.7 (IQR 9.7–not reached) months (≥35 g/L) (*p* < 0.001) (Figure 1B).

### Predicting Survival at 12 Weeks

A small, but a significant proportion [*n* = 54 (14%)] of patients died within 12 weeks of starting treatment (Table 2). In the targeted therapy cohort, 13 (8%) patients died during this time period. Among these patients, 11 (85%) had pre-treatment albumin <35 g/L. The relative risk of death before 12 weeks was 9.58 (95%CI 2.20–41.72, *p* = 0.003) for patients with a pre-treatment albumin <35 g/L compared with those with albumin ≥35 g/L. In the Immunotherapy cohort, 41 (15%) patients died within 12 weeks of starting treatment. Among these patients, 31 (76%) had pre-treatment albumin <35 g/L. The relative risk of death before 12 weeks was 3.6 (95%CI 1.74–6.57, *p* < 0.001) for patients with a pre-treatment albumin <35 g/L compared with those with albumin ≥35 g/L.

**Table 2:** The relationship between pre-treatment albumin and overall survival at 12 weeks, 6 months and 1 year in patients with NSCLC treated with first-line targeted therapy or immunotherapy-based therapy.

	**Albumin**	**Patients**	**Survival at 12 weeks**	**Survival at 6 months**	**Survival at 1 year**
		***n* (%)**	***n* (%)**	***n* (%)**	***n* (%)**
Targeted Therapy	≥35g/L	101 (64)	99 (98)	86 (85)	60 (59)
	<35g/L	58 (36)	47 (81)	40 (69)	28 (48)
Immunotherapy	≥35g/L	120 (52)	110 (92)	99 (83)	59 (49)
	<35g/L	110 (48)	79 (72)	60 (55)	27 (25)

### Prognostic Value of 12-Week Albumin

**Figure 2:**
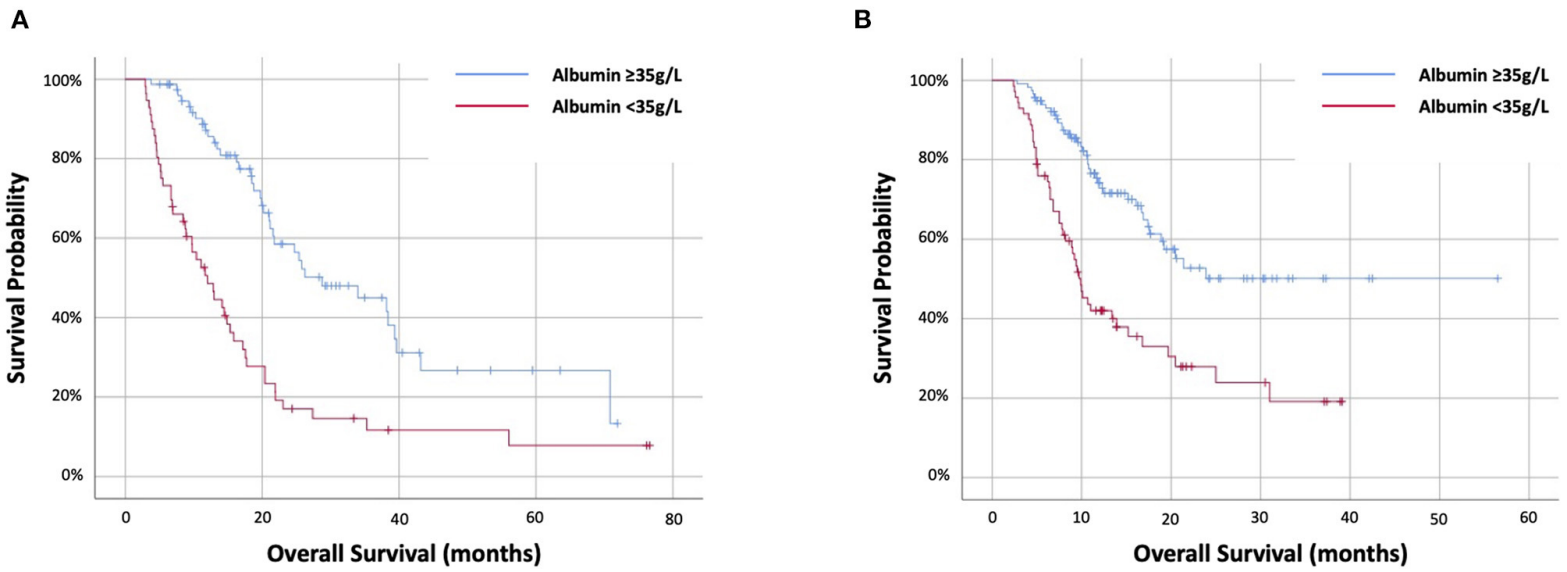
Kaplan–Meier survival curves examing the relationship between 12-week albumin and overall survival of patients with NSCLC treated with first-line **(A)** targeted therapy or **(B)** immunotherapy-based therapy.

The prognostic value of 12-week albumin was evaluated. Data were available for 320 out of 335 (96%) patients alive at 12 weeks, including 133 out of 146 (91%) targeted therapy and 187 out of 189 (99%) immunotherapy cohort. In the targeted therapy cohort, 56 (42%) had 12-week albumin <35 g/L. Twelve-week albumin was predictive of OS at HR2.88 (95%CI 1.86–4.46) (*p* < 0.001) and stratifying OS from 12 (IQR 5.2–20.4) months (<35 g/L) to 28.8 (18.5–70.8) months (≥35g/L) (*p* < 0.001) (Figure 2A). In the immunotherapy cohort, 71 (38%) had 12-week albumin <35 g/L. Twelve-week albumin was predictive of OS at HR2.67 (95%CI 1.74–4.08) (*p* < 0.001) and stratifying OS from 9.9 (IQR 6.2–25) months (<35 g/L) to not reached (IQR 11.8–not reached) months (≥35 g/L) (*p* < 0.001) (Figure 2B).

### Prognostic Value of Dynamic Change of Albumin

The prognostic value of change in albumin status from pre-treatment to 12-weeks was evaluated. A positive dynamic change was defined as an increase in serum albumin from <35 g/L to ≥35g/L at 12-weeks. A negative dynamic change was defined as a decrease in serum albumin from ≥ 35g/L pre-treatment to <35 g/L at 12-weeks.

In the targeted therapy cohort, 46 out of 133 (35%) evaluable patients had a dynamic change in albumin (Table 3). Among these 46 patients, 18 (39%) had a positive change and 28 (32%) among the remaining 87 patients had a negative dynamic change. A positive dynamic change was associated with favourable OS compared with patients whose 12-week albumin remained <35 g/L (*p* = 0.021). A negative dynamic change was associated with poorer OS than those whose 12-week albumin remained ≥35 g/L (*p* < 0.001). However, amongst patients with a 12-week albumin ≥35 g/L, OS was not significantly different between patients with a pre-treatment albumin <35 g/L vs. ≥35 g/L (21.6 vs. 34 months, *p* = 0.458). Similarly, among the patients with 12-week albumin <35 g/L, there was no difference in PFS or OS between patients with a pre-treatment albumin <35 g/L vs. ≥35 g/L (12.9 vs. 10.4 months (*p* = 0.647).

**Table 3:** The relationship between dynamic change in albumin between pre-treatment and 12 weeks and overall survival in patients with NSCLC treated with first-line targeted therapy or immunotherapy-based therapy: univariate log-rank analysis.

**Albumin—Pre-Treatment**	**Albumin −12 weeks**	***n* (%)**	**Overall Survival**	
			**Median (IQR)**	***p***
**Targeted Therapy**				
≥35g/L	≥35g/L	59 (68)	34.0 (18.5–70.8)	***<** **0.001***
≥35g/L	<35g/L	28 (32)	10.4 (4.5–20.4)	
<35g/L	≥35g/L	18 (39)	21.6 (18.7–n/r)	***0.021***
<35g/L	<35g/L	28 (61)	12.9 (6.7–20.4)	
***Immunotherapy***				
≥35g/L	≥35g/L	91 (83)	n/r	***0.129***
≥35g/L	<35g/L	18 (17)	19.7 (6.5–n/r)	
<35g/L	≥35g/L	25 (32)	20.5 (11.7–n/r)	***0.011***
<35g/L	<35g/L	53 (68)	9.2 (5.1–20.5)	

*n/r = not reached. Bold italic values are statistically significant variables*.

In the immunotherapy cohort, only 43 out of 187 (23%) evaluable patients had a dynamic change in albumin, while 25 out of 78 (32%) had a positive change and 18 out of 109 (17%) had a negative dynamic change. A positive dynamic change was associated with favourable OS compared to patients whose 12-week albumin remained <35 g/L (*p* = 0.011). However, among the patients with a 12-week albumin ≥35 g/L, OS was not significantly different between patients with a pre-treatment albumin <35g/L vs. ≥35 g/L (20.5 vs. not reached months, *p* = 0.423).

### Prognostic Value of Albumin at the Time of Progression

The prognostic value of albumin at the time of radiological or clinical progression of disease prompting cessation of treatment was evaluated. Data were available for 274 out of 287 patients with PD, among which 121 out of 128 (95%) were targeted therapy and 153 out of 159 (96%) were immunotherapy cohort.

**Figure 3:**
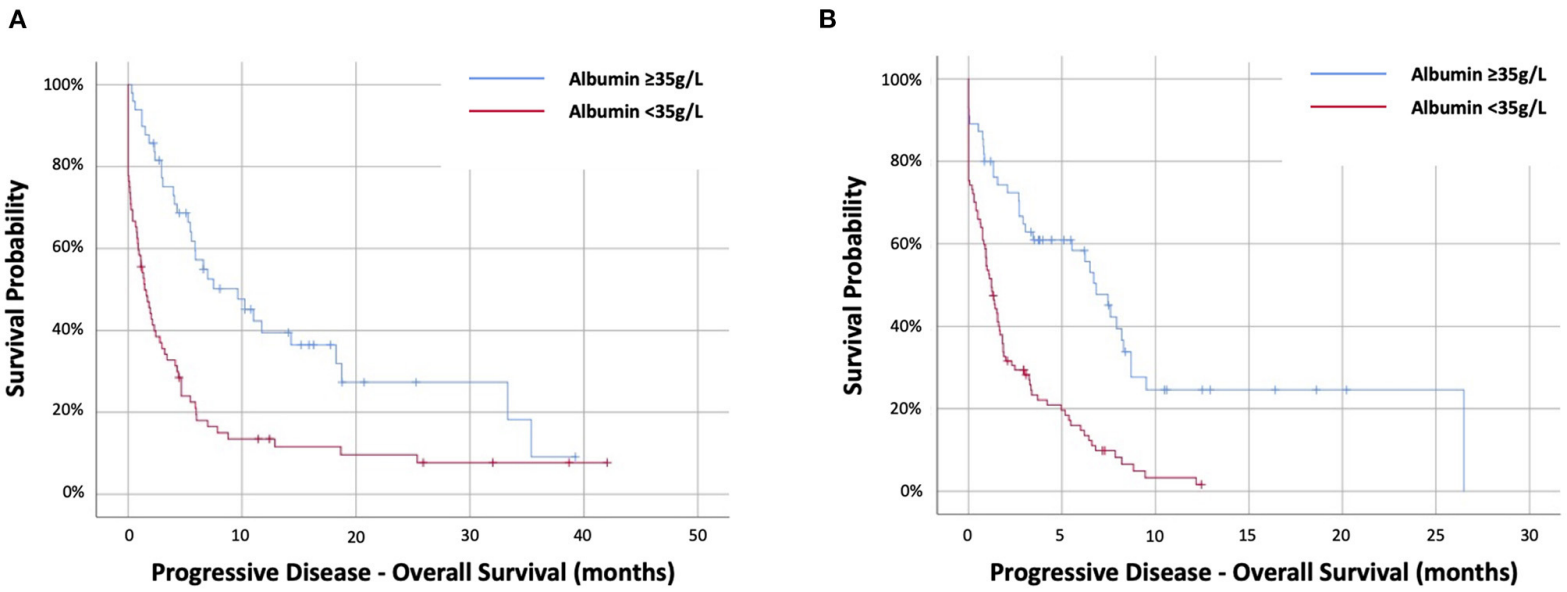
Kaplan–Meier survival curves examing the relationship between progressive-disease albumin and progressive disease overall survival of patients with NSCLC treated with first-line **(A)** targeted therapy or **(B)** immunotherapy-based therapy.

In the targeted therapy cohort, 72 (59%) had PD-albumin <35 g/L. Progressive disease-albumin was predictive of PD-OS at HR2.48 (95%CI 1.61–3.82) (*p* < 0.001) and stratifying PD-OS from 1.4 (IQR.1–4.7) months (<35 g/L) to 9.6 (IQR 4–33.3) months (≥35 g/L) (*p* < 0.001) (Figure 3A). In the immunotherapy cohort, 98 out of 153 (64%) had PD-albumin <35 g/L. Progressive disease-albumin was predictive of PD-OS at HR2.87 (95%CI 1.91–4.31) (*p* < 0.001) and stratifying PD-OS from 1.2 (IQR 0.1–3.4) months (<35 g/L) to 6.8 (IQR 1.6–9.5) months (≥35 g/L) (*p* < 0.001) (Figure 3B).

## Discussion

Biomarkers of systemic inflammation and malnutrition, such as albumin, are firmly established as having a prognostic value in patients with cancer. Hypoalbuminaemia is an important prognostic factor for patients with NSCLC, including in patients with localised disease treated with surgery or those with metastatic disease treated with cytotoxic chemotherapy (22–25, 27–30). This study found that albumin independently predicts survival in patients with metastatic NSCLC treated with either first-line targeted therapy or immunotherapy-based treatments. This is an important finding given the distinct molecular and clinical characteristics of these subtypes of NSCLC. In particular, first-line immunotherapy-based treatment regimens show limited efficacy in patients with actionable mutations (11, 31–33).

The key strength of our study is in the simplicity of utilising a single routine blood test that provided useful prognostic information in well-defined clinically relevant patient groups based on “standard of care” molecularly defined treatment pathways. Although albumin has previously been shown to predict survival in patients with metastatic NSCLC treated with the EGFR targeted therapy erlotinib, only 6.8% of patients had a known EGFR mutation and 90% had previous exposure to other SACT agents (27). Similarly, although several studies had investigated the prognostic value of albumin in patients with NSCLC treated with immunotherapy, many have combined albumin with other factors, and none have restricted assessments to first-line treatment only (34–37).

Despite the suggestion made by the standard pre-treatment assessment that all patients in this study were fit for SACT, more than 10% of patients died within 12 weeks of starting treatment. This timescale is clinically relevant as patients typically undergo first formal assessment of treatment efficacy at this time and expected life expectancy of at least three months was a key eligibility criterion in the clinical trials of these agents (6–8, 38). In both the targeted therapy and immunotherapy cohorts, albumin <35 g/L was associated with a significantly higher risk of death within 12 weeks of starting treatment. A key aim of pre-treatment prognostic biomarkers is to identify patients who will not benefit from treatment to facilitate personalised treatment options and enable realistic discussions with patients about expected outcomes. Specifically, this may reduce the risks associated with treatment and enable appropriate palliative care and end-of-life decision making in a planned manner.

Significantly, few studies had investigated the prognostic value of biomarkers of systemic inflammation beyond pre-treatment assessment. The neutrophil/lymphocyte ratio (NLR) or platelet/lymphocyte ratio (PLR) during or after treatment have been shown to be associated with survival in patients with NSCLC treated with SACT (24, 39–42). An increase in NLR ≥3 from pre-treatment to 6 weeks is associated with poorer outcomes in patients with NSCLC treated with immunotherapy (42). In our study, although dynamic changes of albumin status were associated with differences in survival, the prognostic value of 12-week albumin was independent of pre-treatment albumin. In patients who were alive at 12 weeks, albumin <35 g/L was associated with a worse ongoing prognosis in both cohorts.

These findings also suggested that albumin has ongoing prognostic value during the treatment journey of a patient. An improvement in albumin levels while on treatment may represent better cancer control. Whether this is by reducing cancer activity directly, or indirectly by reducing inflammation is not clear. By extension, declining albumin may represent a loss of cancer control. This may have novel clinically relevant implications. Firstly, serial measurement of albumin, and potentially other biomarkers of systemic inflammation, may be useful as tools for monitoring treatment response. Declining albumin status while on treatment may help identify patients who are developing treatment resistance. In clinical practise, this may support decisions for earlier formal response assessments or timelier switches to alternative therapy. This is strongly supported by our finding that a majority of patients in each cohort had hypoalbuminaemia at the time of progressive disease. Progressive disease-albumin <35 g/L was associated with a median survival of <6 weeks in each cohort. Therefore, these patients were unlikely to derive benefit from second-line therapy, which should not be recommended. To our knowledge, we are the first to assess the prognostic value of biomarkers of systemic inflammation at the time of disease progression in these patient cohorts.

Secondly, these findings strengthened the growing evidence promoting the use of rehabilitation in patients with cancer. Albumin, like other biomarkers of systemic inflammation, reflects the multi-factorial syndrome of cachexia (19, 20). Strategies to stabilise patient fitness and reduce systemic inflammation/cachexia may have important roles alongside SACT in the holistic management of patients with cancer (18, 43). Recently published guidelines in the UK have recommended integrating pre-treatment rehabilitation, i.e., prehabilitation, into established clinical pathways for patients with cancer (44). Interventions to optimise physical functional and nutritional status may be employed based on the needs of the patient (18, 43–46). Patients with metastatic NSCLC often have multiple pre-existing comorbidities and a high cancer-symptom burden (47). In our cohort, 64 (16%) had pre-treatment PS2+, which is consistent with that seen in previous real-world studies (48). Overall, this is a patient group who were likely to require intensive, specialist, multi-modal intervention to maximise their fitness. For example, recent data has suggested that half of the patients with metastatic NSCLC who are fit for SACT meet the criteria for the critical need to see a dietician (47).

Interventional rehabilitation studies for patients with cancer are in progress (45, 49). The use of systemic therapies to improve muscle mass and reduce cachexia are also being trialled, although none have entered routine clinical practise (50, 51). We strongly advocate the evaluation of the impact of rehabilitation interventions on biomarkers of systemic inflammation and the relationship to outcomes in patients with metastatic cancer receiving SACT. In the meantime, we suggested that these biomarkers may have roles to play in selecting patient groups for rehabilitation, stratifying interventions and monitoring response.

## Conclusions

The results of the present study confirmed that albumin is an important prognostic factor in patients with metastatic NSCLC, predicting survival independent of the class of drug treatment based on molecular profiling. Most importantly, we provided new evidence to show that tracking albumin during therapy may reveal information about disease activity and treatment response over time. Further work is needed to confirm whether strategies aimed at improving albumin will lead to improved outcomes in patients with metastatic NSCLC receiving SACT.

## Data Availability Statement

The raw data supporting the conclusions of this article will be made available by the authors, without undue reservation.

## Ethics Statement

Ethical review and approval was not required for the study on human participants in accordance with the local legislation and institutional requirements. Written informed consent for participation was not required for this study in accordance with the national legislation and the institutional requirements.

## Author Contributions

MS and IP conceived the idea. MS, AS, KC, T-ED, JL, FT, and CS led the primary data collection. MS, AS, KC, and T-ED were responsible for data cleaning. MS analysed the data. MS, AS, KC, and IP prepared the draft manuscript. T-ED and MM provided significant intellectual input and advice in the re-draft of the manuscript. All authors approved the final version of the manuscript.

## Conflict of Interest

The authors declare that the research was conducted in the absence of any commercial or financial relationships that could be construed as a potential conflict of interest. The handling editor declared a shared affiliation with several of the authors (MS, CS, FT) at time of review.

## Publisher's Note

All claims expressed in this article are solely those of the authors and do not necessarily represent those of their affiliated organizations, or those of the publisher, the editors and the reviewers. Any product that may be evaluated in this article, or claim that may be made by its manufacturer, is not guaranteed or endorsed by the publisher.
